# *In Vitro* Antiproliferative Effect of *Arthrocnemum indicum* Extracts on Caco-2 Cancer Cells through Cell Cycle Control and Related Phenol LC-TOF-MS Identification

**DOI:** 10.1155/2013/529375

**Published:** 2013-09-30

**Authors:** Mondher Boulaaba, Khaoula Mkadmini, Soninkhishig Tsolmon, Junkyu Han, Abderrazak Smaoui, Kiyokazu Kawada, Riadh Ksouri, Hiroko Isoda, Chedly Abdelly

**Affiliations:** ^1^Laboratoire des Plantes Extrêmophiles, Centre de Biotechnologie de Borj Cédria, BP 901, 2050 Hammam-Lif, Tunisia; ^2^Graduate School of Life and Environmental Sciences, University of Tsukuba, Tennodai 1-1-1, Tsukuba, Ibaraki 305-8572, Japan; ^3^Alliance for Research on North Africa (ARENA), University of Tsukuba, Tennodai 1-1-1, Tsukuba, Ibaraki 305-8572, Japan

## Abstract

This study aimed to determinate phenolic contents and antioxidant activities of the halophyte *Arthrocnemum indicum* shoot extracts. Moreover, the anticancer effect of this plant on human colon cancer cells and the likely underlying mechanisms were also investigated, and the major phenols were identified by LC-ESI-TOF-MS. Results showed that shoot extracts had an antiproliferative effect of about 55% as compared to the control and were characterised by substantial total polyphenol content (19 mg GAE/g DW) and high antioxidant activity (IC_50_ = 40 **μ**g/mL for DPPH test). DAPI staining revealed that these extracts decrease DNA synthesis and reduce the proliferation of Caco-2 cells which were stopped at the G_2_/M phase. The changes in the cell-cycle-associated proteins (cyclin B1, p38, Erk1/2, Chk1, and Chk2) correlate with the changes in cell cycle distribution. Eight phenolic compounds were also identified. In conclusion, *A. indicum* showed interesting antioxidant capacities associated with a significant antiproliferative effect explained by a cell cycle blocking at the G_2_/M phase. Taken together, these data suggest that *A. indicum* could be a promising candidate species as a source of anticancer molecules.

## 1. Introduction

Cell cycle progress is partially controlled by the balance between the accumulation of reactive oxygen species (ROS) and the antioxidant system [1]. A disturbance during the cell division can lead to abnormal cell proliferation. The overproduction of ROS results in oxidative stress, a deleterious process that can be an important mediator of cell structure damages and initiation of serious diseases such as cancer [2]. ROS are implicated in ischemia-induced permeability changes of the intestine, in Crohn's disease, and in ulcerative colitis [3]. Several approaches including apoptosis [4], autophagy [5], and differentiation [6] are used to control and eradicate cancer cells. Recently, the analysis of cell cycle arrest emerged as a novel approach for cancer eradication [7]. Cells recognise and respond to extracellular stimuli by engaging specific intracellular programs, such as the signalling cascade that leads to the activation of mitogen-activated protein kinases (MAPKs) [8]. All eukaryotic cells possess multiple MAPK pathways which coordinately regulate diverse cellular activities like gene expression, mitosis, metabolism, survival, and cell death [9]. The importance of MAPK pathways into cell proliferation and death is highlighted by the observation that deregulation of these kinase cascades can result in cell transformation and cancer [10].

Recently, three groups of mitogen-activated protein kinases (MAPKs) responsible for the extracellular stimuli response cascade were characterised in cells as extracellular signal-regulated kinases (ErKs 1 to 5), c-Jun amino-terminal kinases (JNKs 1, 2, and 3), and p38 isoforms [11, 12]. MAPKs phosphorylate specific serines and threonines of target protein substrates and regulate cellular activities like gene expression, mitosis, movement, metabolism, and programmed death [12]. During cell division, checkpoint controls are characterised by a number of Cdk/cyclin families, which are responsible for the cell cycle progression [13]. Moreover, checkpoint kinases such as Chk1 and Chk2 are responsible for the mechanism involved in the induced cell cycle arrest [14]. The p38 MAPK pathway is implicated in the suppression of tumorigenesis, since it can (1) inhibit the cell growth by decreasing the expression of cyclin D [15], (2) inhibit the activity of Cdc25 phosphatases [16], and (3) engage the p16/Rb and p19^ARF^/p^53^ tumour suppressor pathways [17]. Consequently, the p38 MAPK pathway is activated upon cellular stress and often engages process that can block proliferation (cell cycle arrest) or promote apoptosis. The extracellular signal-regulated kinases (ErKs) with the p38 pathways are all new molecular targets for therapeutic research [12]. MAP kinases inhibitors will certainly be the next developed mediators for the treatment of human disease [12].

The therapeutic effects of plants have been partly ascribed to their high content in bioactive molecules, such as phenolic compounds. To overcome oxidative stress generated by severe habitat conditions, plants produce these metabolites. This is the case of several halophyte species, which are used in folklore medicine, since the extracts proved to have activity against human, animal, and plant pathogens [18]. Phenols are an integral part of human diet due to their abundance in fruits and vegetables and have attracted considerable interest as powerful antioxidant compounds [19]. Besides, these compounds present large biological properties such as antimicrobial, antiviral, anti-inflammatory, antiallergic, antithrombotic, cardioprotective and vasodilatory effects [20]. For example, flavonoids, flavonolignans, isothiocyanates, and proanthocyanidins are known to play an important role in the cancer cell behaviour [18]. The protective effect of polyphenols from *Glycyrrhiza glabra* extracts against oxidative stress in human cancer cells was also documented [21].

*Arthrocnemum indicum* is a traditional medicinal halophyte common as salt marshes in Tunisia [18]. It is used in the treatment of poisonous snakebites and scorpion stings. It also plays a prominent role in traditional oriental medicine and ancient Indian medicine (Ayurveda). Here, we assessed mainly the effect of *A. indicum* shoot extracts on the human colon cancer Caco-2 cell proliferation through the control of the cell cycle. Caco-2 cell lines are frequently used as a model in order to study the anticancer effect [22], the inhibition of inflammatory mediators [23], and the degree of transepithelial resistance [24]. The present study aims at evaluating the effect of shoot extracts of the halophyte *A. indicum* on Caco-2 cell growth. The possible underlying mechanisms involving Erk1/2 and p38 MAP kinases on cell cycle arrest were investigated, and the major phenolics in shoot extracts were identified by LC-ESI-TOF-MS.

## 2. Materials and Methods

### 2.1. Sampling and Sample Preparation

*A. indicum *(Chenopodiaceae) shoots were harvested at full flowering stage from the sebkha of El Kelbia locality (20 km northeast Kairouan; superior semiarid bioclimatic stage; mean annual rainfall: 400 mm) in May 2010. The harvested shoots were rinsed with distilled water, left at room temperature for 7 days in the dark, and ground to fine powder. For the anticancer effect analysis, extracts were obtained by soxhlet extraction at a ratio of 20 g dry powder in 200 mL of 80% methanol. They were kept for 24 h at 4°C, filtered through a Whatman n°4 filter paper, evaporated under vacuum, and were stored at 4°C until analysis. For the anticancer effect analysis, 10 g of powder was added to 100 mL 80% methanol, stored for one week at room temperature in the dark, and then filtered through a Millipore filter (0.2 *μ*m). After drying under vacuum, the powder was dissolved in DMSO to get 2.5% (2.5 mg powder in 100 *μ*L DMSO) as stock concentration. Finally, extracts were stored at −80°C until analysis.

### 2.2. Quantification of Phenolic Fractions

- Total polyphenols: Folin-Ciocalteu reagent was used to determine the amount of total phenolics in methanolic extracts [19]. An aliquot of 125 *μ*L of diluted extract was added to 500 *μ*L of distilled water and 125 *μ*L of the Folin-Ciocalteu reagent. The mixture was shaken before adding 1250 *μ*L of Na_2_CO_3_ (7%) and adjusted with distilled water to a final volume of 3 mL. After incubation for 90 min at 23°C in the dark, the absorbance versus prepared blank was read at 760 nm. Total phenolic content was expressed as mg gallic acid equivalent per gram of dry weight (GAE/g DW) using a calibration curve with gallic acid, ranging from 0 to 500 *μ*g/mL. All samples were analyzed in triplicate.
- Total flavonoids: the measurement of flavonoid content in *A. indicum* shoots was based on the method described by Ksouri et al. [19]. An aliquot of the samples or (+)-catechin standard was added to test tubes containing 75 *μ*L of a 5% NaNO_2_ solution and mixed for 6 min. Then, 150 *μ*L of 10% AlCl_3_ solution was added. After 5 min at room temperature, 500 *μ*L of 1 N NaOH was added. The final volume was adjusted to 2.5 mL with distilled water and thoroughly mixed. Absorbance of the mixture was determined at 510 nm against the blank where the sample was omitted. Total flavonoid content was expressed as mg catechin equivalent per gram of dry weight (mg CE/g DW), through the calibration curve of (+)-catechin, ranging from 0 to 500 *μ*g/mL. All samples were analyzed in triplicate.
- Total condensed tannins: the content of condensed tannin was determined according to Ksouri et al. [19]. Briefly, 50 *μ*L of diluted shoot extracts was mixed with 3 mL of 4% vanillin solution in methanol and 1.5 mL hydrochloric acid (1 N). The mixture was allowed to stand for 15 min, and the absorbance was measured at 500 nm against 80% methanol. Results were expressed as mg catechin equivalent per gram of dry weight (mg CE/g DW). All samples were analyzed in triplicate.

### 2.3. Determination of Antioxidant Assays

(i) Evaluation of total antioxidant capacity: an aliquot (100 *μ*L) of plant extract was added to 1 mL of reagent solution (0.6 M sulfuric acid, 28 mM sodium phosphate, and 4 mM ammonium molybdate). The tubes were then incubated at 95°C for 90 min. After the mixture has been cooled at room temperature, the absorbance was measured at 695 nm (Anthelie Advanced 2, SECOMAN) against a blank. The total antioxidant activity was expressed as mg GAE/g DW [19]. The calibration curve was established between 0 and 500 *μ*g/mL. All samples were analyzed in triplicate.
(ii) Stable free radical scavenging capacity: DPPH (1,1-diphenyl-2-picrylhydrazyl) quenching ability of plant extracts was measured according to Ksouri et al. [19]. One milliliter of the extract at different concentrations was added to 250 *μ*L of a 2 mmol/L DPPH methanol solution. The mixture was shaken vigorously and then left at room temperature for 30 min in the dark. The absorbance of the resulting solution was then measured at 517 nm and corresponded to the ability of extracts to reduce the stable radical DPPH to the yellow-colored diphenylpicrylhydrazine. The extract concentration required to cause a 50% inhibition is expressed as IC_50_ (*μ*g/mL) and calculated using the following equation:
  (1) DPPH  scavenging  effect  (%)  =  [A0−A1A0]∗100,
  where *A*_0_ is the absorbance of the control at 30 min and *A*_1_ is the absorbance of the sample at 30 min. All samples were analyzed in triplicate
(iii) Iron reducing power: the iron (III) reductive capacity of the extract was assessed as described by Ksouri et al. [19]. Briefly, 1 mL of methanol extract was mixed with 2.5 mL phosphate buffer (0.2 mol/L, pH 6.6) and 2.5 mL K_3_Fe (CN)_6_ solution (1 g/100 mL). After 20 min at 50°C, 2.5 mL trichloroacetic acid (10 g/100 mL) was then added, and the mixture was centrifuged for 10 min at 650 ×g. Finally, the upper layer fraction (2.5 mL) was mixed with 2.5 mL of ultrapure water and 0.5 mL of ferric chloride (0.1 g/100 mL). Absorbance was measured at 700 nm. Ascorbic acid was used as a positive control. The higher absorbance indicates a higher reducing power. EC_50_ value (*μ*g/mL) is the effective concentration giving an absorbance of 0.5 for reducing power and was obtained from linear regression analysis. All samples were analyzed in triplicate.

### 2.4. Cell Maintenance

The human carcinoma Caco-2 cell line was isolated from the colon cancer of a 72-year old Caucasian male. Culture was maintained in Dulbecco's modified Eagle's medium (DMEM, Sigma) supplemented with 10% heat-inactivated foetal bovine serum (FBS, Sigma), 1% nonessential amino acids (Cosmo Bio Co., LDT), and 1% penicillin (5000 IU/mL)-streptomycin (5000 *μ*L/mL) solution (ICN Biomedicals) at 37°C under 5% CO_2_ atmosphere.

### 2.5. Antiproliferative Effect by 3-(4,5-Dimethylthiazol-2-yl)-2,5-diphenyltetrazolium Bromide (MTT) Assay

To investigate the antiproliferative effect, Caco-2 cells were seeded in 96-well plates at a concentration of 2 × 10^4^ cells/mL in Dulbecco's modified Eagle's medium (DMEM). Cells were kept at 37°C under 5% CO_2_ and treated with different concentrations of DMSO. *A. indicum *shoot extracts ranged between 0.01 and 100 *μ*g/mL. After 72 h of treatment, 10 *μ*L MTT solution (5 mg/mL) was added to the culture medium. After 24 h of incubation, the formazan produced was dissolved using 100 *μ*L of 10% SDS solution (Wako). Absorbance was measured at 570 nm on a multidetection microplate reader [6]. Results shown represent the mean of three independent experiments.

### 2.6. DAPI Staining

During the analysis by fluorescence microscopy, cells (2 × 10^4^ cells/mL) were incubated for 72 h with 100 *μ*g/mL *A. indicum* shoots. Control cells were treated with 0.4% DMSO. Then, samples were washed 2 times with PBS and fixed with 3.7% formaldehyde in PBS for 10 min at room temperature. After washing two times with PBS and staining with DAPI (4,6-diamidino-2-phenylindole) solution, the chromosomes were analyzed [25]. Results represent the mean of three independent experiments.

### 2.7. Cell Cycle Analysis

The cell cycle analysis was assessed using guava flow cytometry (Guava Technologies). To determine the plant effect on the cancerous cell division, Caco-2 cells (2 × 10^4^ cells/mL) were pretreated for 72 h with 100 *μ*g/mL* A. indicum* extracts. Then, cells were washed with PBS, fixed with 70% ice-cold ethanol, and stored at −20°C until analysis. After removing ethanol, the cells were suspended in 500 *μ*L of cell cycle reagent (Guava Technologies) and incubated in the dark at room temperature for 30 min [6]. The results represent the mean of three independent experiments.

### 2.8. Western Blotting

To evaluate the effects of *A. indicum* extracts on the expression of checkpoint protein kinases, 2 × 10^4^ cells/mL were seeded for 72 h in culture dish with 100 *μ*g/mL of *A. indicum* shoot extracts. The treated cells were washed with PBS and lysed by RIPA buffer (Sigma Aldrich Co.) with protease inhibitor cocktail (Sigma Aldrich Co.). The mixture was centrifuged at 12,000 × g for 20 min at 4°C. The protein-containing supernatant was kept, and the quantification of the proteins was performed using the Plus One 2D Quant kit (GE Healthcare). Proteins (20 *μ*g) were resolved on 12% sodium dodecyl sulfate-polyacrylamide gel electrophoresis and transferred onto nitrocellulose membrane using the iBlot dry blotting system (Invitrogen). After blocking with 5% nondry fat milk, the membrane was incubated at 4°C overnight under shaking with the appropriate antibodies. The bands of cyclin B1, Erk1/2, pErk1/2, p38, pp38, *β*-actin, Chk1, pChk1, Chk2, and pChk2 proteins were detected by horseradish peroxidase-conjugated secondary antibodies using the enhanced chemiluminescence system ECL (Amersham Biosciences). After band staining, the gels were scanned and converted to images and then analyzed with ImageJ software (GE Healthcare). The images were rectified and transformed into binary images to calculate the relative density estimated as a percentage of band appearance. Results represent the mean of three independent experiments.

### 2.9. Analysis of *A. indicum* Shoot Extracts by Liquid Chromatography/Electrospray Ionization Time-of-Flight Mass Spectrometry or LC/ESI-TOF-MS

The extracts obtained by soxhlet extraction were kept. The methanolic phase was passed through C_18_ column to eliminate chlorophyll and nonpolar compounds. The sample was then passed through a 0.45 *μ*m nylon filter before the injection into the LC-ESI-TOF-MS system. Chromatographic and mass spectrometer conditions: the separation of selected phenolic compounds was carried out using an HPLC system (Agilent 1200, Agilent technologies, Germany) equipped with a reversed phase C_18_ analytical column (2.5 × 50 mm) and 1.8 *μ*m particle size (Zorbax Eclipse XDB-C_18_). The mobile phase B was milli-Q water consisted of 0.1% formic acid. The mobile phase A was acetonitrile. This HPLC system was connected to a time-of-flight mass spectrometer, Agilent MSD TOF (Agilent technologies, Germany), equipped with an electrospray interface operating in positive and negative modes. In this study, some parameters were used in order to increase the possibilities of separation, detection, and characterization of phenolic compounds that are responsible for the biological activities. About the chromatographic conditions: the column temperature was maintained at 23°C, the flow rate of the mobile phase was 0.4 mL/min, and the injected sample volume was 2 *μ*L. The optimised gradient elution was illustrated as follows: 0–10 min, 10–20% A; 10–15 min, 20–30% A; 15–25 min, 30–50% A; 25–35 min, 50–70% A; 35–40, 70–80% A; 40–65 min, which return to initial conditions. Concerning MS conditions, the capillary voltage was 3500 V, the nebuliser pressure 30 psig, drying gas 8 l/min, gas temperature 325°C, fragmentor voltage fragment 175 V, skimmer voltage 65 V, and octopole RF 750 V. LC/MS accurate mass spectra were recorded across the range 100–3000 *m*/*z*. Electrospray ionization is operated in positive mode. The data recorded was processed with MassHunter software (Germany) with accurate mass application of specific additions from Agilent MSD TOF software. UV absorption spectra were recorded online during the HPLC analysis. The DAD detector was set to a scanning range of 200–400 nm. The phenolic compounds were identified mainly by their UV data, ESI-MS spectra, and by comparing with published data.

### 2.10. Statistical Analysis

For all plant parameters, all samples were analyzed in three replications. Data are shown as mean ± sd. A one-way analysis of variance (ANOVA) using the post hoc analyse with Duncan's test was carried out to test any significant differences at *P* < 0.05.

## 3. Results 

### 3.1. Phenolic Contents and Antioxidant Activities of *A. indicum *Shoots

Total polyphenolic, flavonoid, and condensed tannin contents of *A. indicum *shoot extract at 18 mg/mL were estimated at the flowering stage. The evaluation of the antioxidant capacities of *A. indicum* shoots was determined by the antiradical activity against DPPH radical, the total antioxidant activity and the Fe-reducing power (Table 1). The phenolic compound content amounted at 19.97 mg GAE/g DW, whereas flavonoid and condensed tannin contents were 11.12 and 1.8 mg CE/g DW, respectively (Table 1). The total antioxidant activity (130 mg GAE/g DW) and the antiradical ability to quench the DPPH radical (IC_50_ = 40 *μ*g/mL) of the shoot extracts were high and concomitant with a moderate Fe-reducing power (EC_50_ = 290 *μ*g/mL). 

### 3.2. Antiproliferative Effect of *A. indicum *Shoots on Caco-2 Cancer Cells

**Figure 1:**
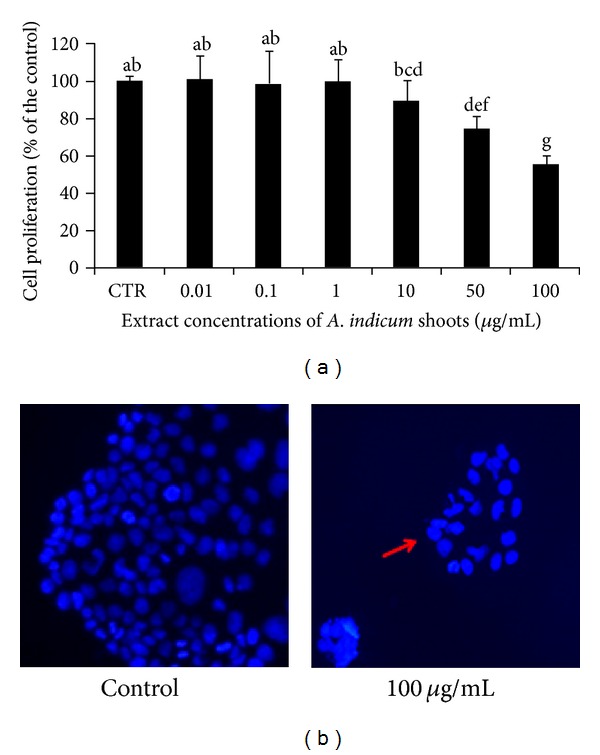
(a) Measurements of cell proliferation using MTT assay in human carcinoma Caco-2 cell line treated with *A. indicum* shoot extracts. Cells at 2 × 10^4^ cells/mL were left untreated or were treated with 0.01, 0.1, 1, 10, 50, or 100 *μ*g/mL of *A. indicum* for 72 h. Values represent the results of three independent experiments. (b) DAPI staining of Caco-2 cells treated with *A. indicum*. Cells at 2 × 10^4^ cells/mL were incubated with 100 *μ*g/mL of *A. indicum* for 72 h. Control cells were incubated with 0.4% DMSO. After 72 h, the nuclear morphologies of cells were examined using a fluorescent DNA-binding agent, DAPI. The DNA was analyzed using fluorescence microscopy. The arrow indicates mitotic cells with chromatin distribution. Results shown (a) and (b) are typical of 3 independent experiments.

The strong accumulation of phenolic compounds in* A. indicum* may confer to this species a strong antiproliferative activity. This potential effect was evaluated using the Caco-2 colon adenocarcinoma cells. *A. indicum* shoot extracts inhibited the Caco-2 colon cancer cell growth in a dose-dependent manner (Figure 1(a)). At low concentrations (0.01–1 *μ*g/mL), no significant effect on Caco-2 cell growth was observed. Whereas, from the concentration of 10 to 100 *μ*g/mL, the plant extract significantly inhibited the growth of Caco-2 cells as compared to the control one. Besides, the most reduction of Caco-2 cell proliferation was about 55% using the high extract concentration (100 *μ*g/mL). 

To further assess whether the antiproliferative activities of extracts on Caco-2 cells were related to the DNA synthesis, the presence of chromatin condensation was analyzed by fluorescent microscopy using the DNA-binding fluorescent dye (DAPI) (Figure 1(b)). Control cells displayed nuclei with homogeneous chromatin distribution, whereas the shoot extracts at 100 *μ*g/mL reduced significantly the DNA synthesis. However, no significant apoptotic effect was observed.

### 3.3. Effect of Shoot Extraction on Cell Cycle Arrest Using Flow Cytometry

Shoot extract at 100 *μ*g/mL affected the cell cycle distribution (Table 2). The G_0_/G_1_ and sub-G_0_ phases showed stable percentages, whereas the S-phase percentage decreased from 17.65% in the control to 14.30%. Interestingly, the percentage of Caco-2 cells at the G_2_/M phase was slightly higher after incubation with *A. indicum* extracts (42.2%) as compared to the control (38.95%). 

### 3.4. Effect of *A. indicum* on the Mitogen-Activated Protein Kinases Involved in G_2_/M Arrest

**Figure 2:**
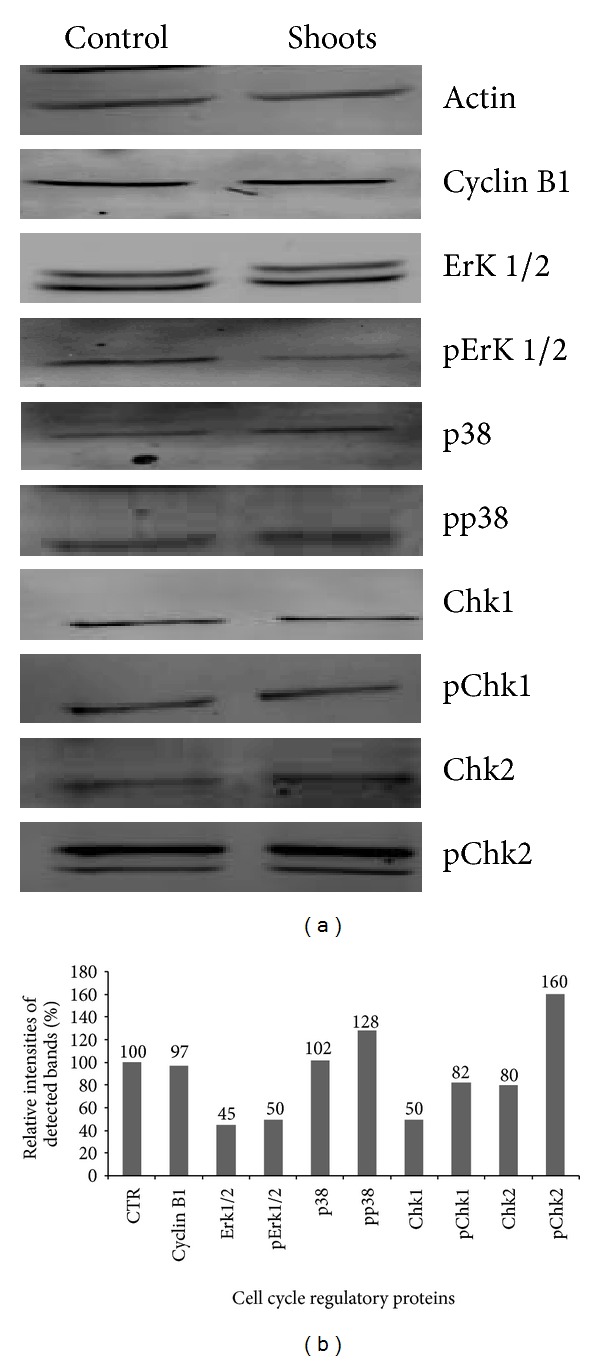
(a) Western analysis of the cell cycle regulatory proteins. Cells at 2 × 10^4^ cells/mL were treated with 100 *μ*g/mL of *A. indicum* for 72 h. After protein extraction, the same blot was incubated with the appropriate antibodies. The analyzed MAP kinases were cyclin B1, Erk1/2, pErk1/2, p38, pp38, Chk1, pChk1, Chk2, and pChk2. Results shown are typical of 3 independent experiments. (b) Relative intensities of detected bands of cyclin B1, Erk1/2, pErk1/2, p38, pp38, Chk1, pChk1, Chk2, and pChk2.

The expression of Erk1/2 and p38 MAP kinases, cyclin B1 and checkpoint kinase proteins (Chk1 and Chk2) in Caco-2 cancer cells were investigated following 72 h exposure to *A. indicum* extract (Figure 2(a)). Moreover, the relative intensities of detecting the bands of the analyzed MAP kinases were shown in Figure 2(b). The cyclin B1, protein was downregulated as compared to the control, whereas the ErK protein expression level was moderately increased by the extract. In contrary, the phosphorylated form was clearly down-regulated. The activation of Erk1/2 protein in cells treated with shoot extracts decreased as compared to the control. In fact, data showed that the ratio pErk/total Erk in cells treated by *A. indicum* dropped as a consequence of the inhibition of the activated form of Erk (pErk). Levels of regulation that contribute to stopping cell division and which involve different MAP kinases are summarised in Figure 2(b). In this context, the crude shoot extract of *A. indicum* appeared to have an upregulating effect on the level of pp38 protein expression as compared with the p38 MAP kinase. This is responsible for the downregulation of the cyclin B1 via the Cdc25c. Furthermore, the treatment with *A. indicum* extracts decreased the expression of the checkpoint kinases Chk1 and Chk2 unlike to what occurred for pChk2. The phosphorus initially attached to the Erk is transmitted to Chk1 and Chk2 MAP kinases which induce the inactivation of the CDK1/cyclin B1 complex. This is the last level of the cell cycle regulation shown after treatment. 

### 3.5. LC-TOF-MS Identification of Bioactive Metabolites in Shoot Extracts of *Arthrocnemum indicum*

**Figure 3:**
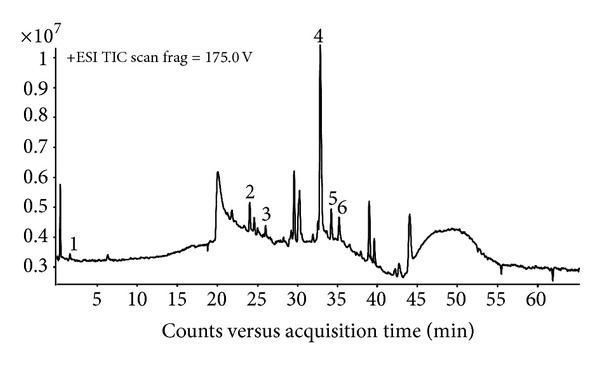
Total ions chromatogram (TIC) of *A. indicum *methanolic extract obtained by LC-ESI-TOF-MS. Peaks designation: (1) gallic acid, (2) 3-hydroxy-4′-methoxyflavone, (3) cyanidin, (4) chrysoeriol, (5) quercetin, (6) catechol, syringic acid, and luteolin.

**Figure 4:**
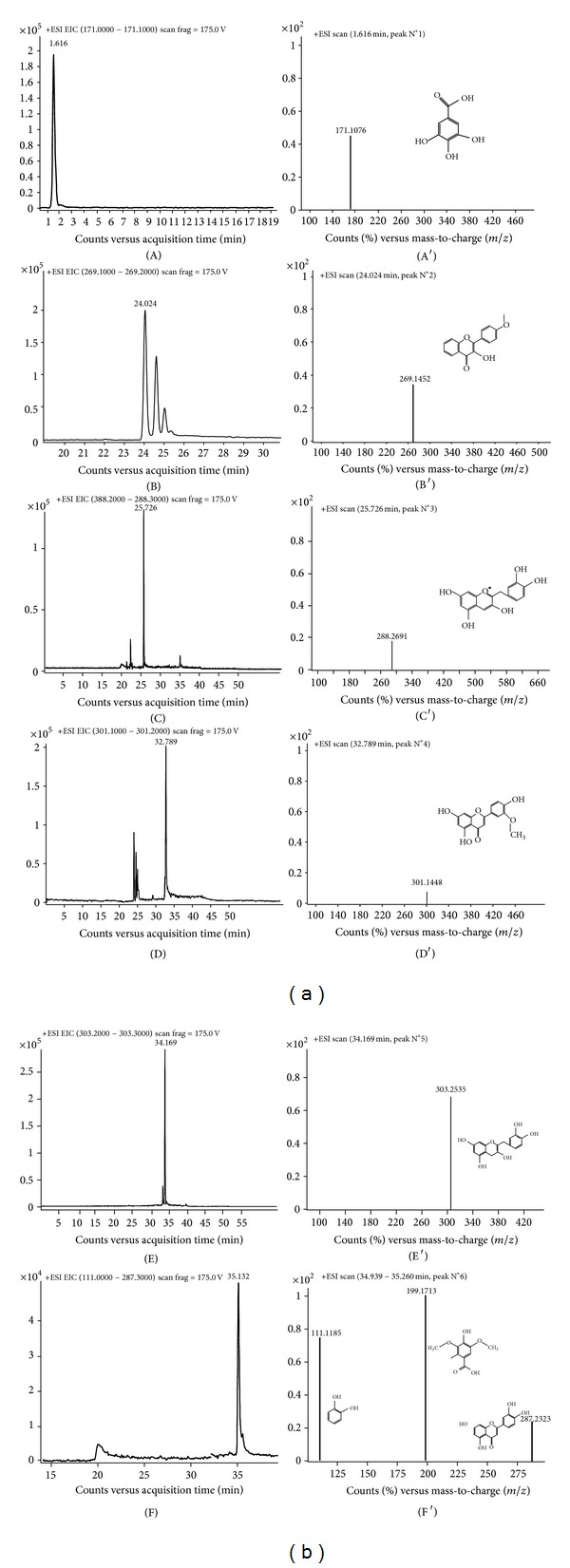
Extract ions chromatogram/mass spectra of each protonated molecule (positive mode): gallic acid ((A)/(A′)), 3-hydroxy-4′-methoxyflavone ((B)/(B′)), cyanidin ((C)/(C′)), chrysoeriol ((D)/(D′)), quercetin ((E)/(E′)), and catechol, syringic acid, and luteolin ((F)/(F′)).

The analysis of the methanolic extracts of *A. indicum* by LC-ESI-TOF-MS in positive mode revealed that this halophyte plant is rich in phenolic compounds. Eight compounds were characterised and further identified by referring to the literature reporting their occurrence in the *Chenopodiaceae* family. The obtained total ion chromatogram (TIC) is illustrated in Figure 3. Furthermore, the extracted ion chromatogram (EIC) and mass spectrum (MS) of each phenolic compound are shown in Figure 4. The analysis showed the strong antioxidant activity of *A. indicum* shoot extracts. Five flavonoid compounds were identified (Figures 4(A′) to 4(F′)): 3-hydroxy-4′-methoxyflavone (*m*/*z* = 269,1452), cyanidin (*m*/*z* = 288,2690), chrysoeriol (*m*/*z* = 301,1448), quercetin (*m*/*z* = 303,2535), and luteolin (*m*/*z* = 287,2202). Moreover, shoots of *A. indicum* accumulate two phenolic acids, namely, gallic (*m*/*z* = 171,1076) and syringic (*m*/*z* = 199,1697) acids. Catechol (*m*/*z* = 111,1173) was also detected with LC-TOF-MS. All these phenolic compounds mentioned are summarised in Table 3, with their molecular formula, selected ion [M + H]^+^, retention time (*R*_*t*_), and UV data of each compound.

## 4. Discussion

### 4.1. Phenolic Contents and Antioxidant Activities of *A. indicum *Shoots

During the past decades, there was an increasing interest in traditional medicine and herbal products. Interestingly, extremophile plants such as halophytes appear to be useful in term of biological activity due to their substantial content in bioactive substances. This was confirmed by the present study on *A. indicum *shoot extracts since the values of total polyphenolic, flavonoid, and condensed tannin contents found were relatively high. The high content of natural polyphenol was already mentioned in tissues of the halophytes* Mesembryantimum* and *Limoniastrum* species [18]. With respect to flavonoids and tannins, these compounds contribute significantly to the total antioxidant activity of many fruits such as red grapes, vegetables, and medicinal plants such as *Nigella sativa* [26]. Shoot extracts were also characterised by an important antioxidant capacity especially against the free DPPH radical at the flowering stage. This could be partly ascribed to the strong accumulation of phenolic compounds during this specific developmental stage. Ksouri et al. [19] reported that *Tamarix gallica* flower extracts had an important total polyphenol content (135 mg GAE/g DW) which was associated with significant antiradical activity and Fe-reducing power (IC_50_ and EC_50_ values were about 2 and 45 *μ*g/mL, resp.).

### 4.2. The Antiproliferative Effect of *Arthrocnemum indicum *Shoots

The antiproliferative effect of various concentrations of polyphenolic extracts was assessed on Caco-2 cell line. Cell proliferation was inhibited in a dose-dependent manner, the optimal concentration of the extract amounting to 100 *μ*g/mL. The anticancer effect of natural products was already demonstrated on different cancer cell lines. For instance, Ren et al. [27] showed the antiproliferative effect of the acetone (AEL) and methanolic (MEL) extracts from *Lethariella zahlbruckneri *on HT-29 human colon cancer cells. Both extracts of *L. zahlbruckneri* decreased viable cell number in dose- and time-dependent manners. With respect to Caco-2, phenolic compounds from apple fruit extracts (with or without skin) inhibited the proliferation of this cell line in a dose-dependent manner [21]. 

In order to better understand the effect of shoot extracts on cell division, the presence of chromatin condensation was analysed by fluorescence microscopy using the DNA-binding fluorescent dye DAPI. This method was already used to demonstrate the cytotoxicity and the apoptosis effects of crude extracts of *Euchresta formosana* radix in the human hepatocellular carcinoma Hep3B cell line [28]. In our study, the control cells showed nuclei with homogeneous chromatin distribution, whereas treatment with 100 *μ*g/mL of extracts decreased the chromatin amount. Hence, shoot extracts had a marked effect on the DNA synthesis. The decrease of DNA biosynthesis which is an indicator for the decrease of the number of cells during treatment provides another argument for the antiproliferative effect of *A. indicum* shoot extracts on Caco-2 cells. In the present study, no apoptosis effect could be observed using the fluorescence microscopy by DAPI staining. Therefore, further experiments like the investigation of the cell cycle distribution are needed to highlight the mechanisms involved in the anticancer activities of those compounds.

### 4.3. Effect of Shoot Extraction on Cell Cycle Arrest Using Flow Cytometry

Given that cell division control is the major regulatory mechanism of cell growth, the analysis of the cell cycle is a novel and relevant approach for cancer control and eradication [7]. Our findings showed that Caco-2 cells were blocked at the G_2_/M phase following 72 h exposure to *A. indicum* shoot extracts at 100 *μ*g/mL. This was already observed in chronic myeloid leukemia (K562) cells treated with *Stellera chamaejasme* extract [6]. The same effect on K562 cells was also reported using a novel and synthetic anticancer agent, the enediyne derivative THDA [29]. Moreover, the role of flavonoid compounds such as 2′-nitroflavone was mentioned for a similar effect on HeLa human cervical carcinoma cells [30].

### 4.4. Effect of Proteins Involved in the G_2_/M Arrest

Cell division is a complex phenomenon that is regulated by a number of protein kinases whose role is the transcription of genes essential for entry into division. These mitogen-activated proteins or MAP Kinases play an essential role in the initiation, the progression, and the coupling of these phases [13]. Our study which aimed at better understanding of the molecular mechanisms of G_2_/M phase arrest induced by *A. indicum* showed that extracts at 100 *μ*g/mL blocked the Caco-2 cell cycle at G_2_/M phase. Moreover, *A. indicum* had an effect on the expression of specific cell cycle-associated protein kinases (cyclin B1, p38, Erk1/2, Chk1, and Chk2) occurring together with the changes in cell cycle distribution. *A. indicum *shoot extracts have a downregulating effect on the expression of cyclin B1 protein. It is assumed that Cdc25c plays a role in the regulation of the Cdk1/cyclin B1 complex [16]. Shoot extracts of *A. indicum* had an upregulating effect on the level of pp38 protein but not on the pErk one. The role of p38 as a key protein in the regulation of cell division was already mentioned in previous studies as an important protein implicated in the suppression of tumorigenesis [15, 17]. The expression of checkpoint kinases Chk1 and Chk2 was also affected, both of these proteins being involved in the anticancer effect in relation with the cell cycle arrest [14]. Thus, all these analyzed protein expressions show for the first times a mechanism related to Caco-2 cancer cells in response to *A. indicum* treatment. The identification of such biological compounds from this plant is needed to clarify the biological effect of this medicinal plant.

### 4.5. LC-TOF-MS Identification of Bioactive Secondary Compounds

In the *Chenopodiaceae* family, some phenolic compounds are abundant. In the present study, gallic acid, 3-hydroxy-4′-methoxyflavone, cyanidin, chrysoeriol, quercetin, catechol, syringic acid, and luteolin were characterised from the aerial parts of *A. indicum*. At 1.616 min (Figure 4(A)), the ion [M + H]^+^ found in positive mode, likely, corresponds to gallic acid with a molecular formula C_7_H_6_O_5_ (Figure 3, Peak no. 1) [31]. At 24.024 min (Figure 4(B)), ion 3-hydroxy-4′-methoxyflavone with the formula C_16_H_12_O_4_ (Figure 3, Peak no. 2) was already found in some African plants [32]. Cyanidin (Figure 3, Peak no. 3) was identified with the molecular formula C_15_H_11_O_6_ detected by the LC-TOF/MS at 25.726 min (Figure 4(C)). However, the compound detected at 32.789 min (Figure 4(D)) was identified as chrysoeriol with the molecular formula C_16_H_12_O_6_ [33] (Figure 3, Peak no. 4). Also, molecular ion detected at 34.169 min (Figure 4(E)) corresponds to quercetin C_15_H_10_O_7_ (Figure 3, Peak no. 5) [34]. As shown in the TIC (Figure 3, Peak no. 6), three ions were detected at 34.939–35.260 min (Figure 4(F)). As a function of previous studies, the first one corresponds to catechol (C_6_H_6_O_2_) [35], the second identified as syringic acid (C_9_H_10_O_5_) [36], and the last one represents luteolin (C_15_H_10_O_6_) according to Liu et al. [37]. 

*A. indicum* shoot extracts produced high antioxidant activities and high antiproliferative activities. This may be explained by the nature of *A. indicum* phenolic amounts. The antioxidant effects of some natural bioactive molecules found in shoot extracts of *A. indicum* as syringic acid, chrysoeriol, and quercetin were already demonstrated in previous studies [38, 39]. Moreover, the methoxyflavonoid and chrysoeriol, selectively inhibit the formation of a carcinogenic estrogen metabolite in MCF-7 breast cancer cells [40]. In addition, luteolin, quercetin, and gallic acid were known by their significant antiproliferative effect [4, 41, 42]. According to Zhang et al. [43], flavonoids such as flavones (luteolin) and flavonols (quercetin) have an important cytotoxic effect on human oesophageal adenocarcinoma cell line (OE33) inducing a cell cycle arrest at the G_2_/M phase. The antioxidant and antiproliferative effects of *A. indicum *extracts could also be explained by the possibility of synergy between components. This has already been demonstrated using HL-60 cells treated with Tunisian Gerboui olive leaf extracts [44].

In conclusion, *A. indicum* may be useful as a candidate in the treatment of the colon cancer in a specific manner. In fact, the high anticancer and antioxidant activities found in shoots of this halophyte could be ascribed to the high total polyphenol content, whereas the significant antiproliferative effect could be explained by the cell cycle arrest on G_2_/M phase determined by flow cytometry. These activities seem to be related to the accumulation of phenolic compounds in *A. indicum*. In this context, eight metabolites were characterised by LC-TOF-MS analysis. 

## Figures and Tables

**Table 1:** Phenolic contents and antioxidant activities of *A. indicum *shoot extracts. The antioxidant activities of extracts were evaluated using total antioxidant activity, antiradical activity as well as the capacity of the extract to reduce the Fe^3+^.

Phenolic contents	Total polyphenols	Total flavonoids	Condensed tannins
	(mg GAE/g DW)	(mg EC/g DW)	(mg EC/g DW)
	19.08	11.12	1.8

Antioxidant activities	Total antioxidant activity	Antiradical activity	Reducing power
	(mg GAE/g DW)	(IC_50_ *µ*g/mL)	(EC_50_ *µ*g/mL)
	130	40	290

**Table 2:** Effect of shoot extracts of *A. indicum* on the cell cycle arrest. Cells were treated with shoot extracts of *A. indicum *at the concentration of 100 *µ*g/mL in order to check the cell cycle distribution. Cells were incubated in the absence (control) and presence of plant extracts during 72 h and then were analyzed by flow cytometry.

	Control	Shoots
G_0_/G_1_	42.4 ± 7.07	42.05 ± 5.16
S	17.65 ± 2.05	14.3 ± 2.69*
G_2_/M	38.95 ± 5.02	42.2 ± 7.5*
Sub-G_0_	1.05 ± 0.07	1.45 ± 0.21

The treatment time is 72 h. Data of three independent experiments are presented as mean ± sd.

*Statistical significance (*P* < 0.05) between treated and control cells.

**Table 3:** Bioactive secondary metabolites determined by HPLC-ESI-TOF-MS in a methanol extract of *A. indicum *shoots.

Peaks	*R*_*t*_	*λ* max	[M + H]^+^	Compounds	Molecular formula
	(min)	(nm)	(*m*/*z*)	(tentatively identified)	
1	1.616	280, 210	171.1076	Gallic acid	C_7_H_6_O_5_
2	24.024	280, 255	269.1452	3-Hydroxy-4′-methoxyflavone	C_16_H_12_O_4_
3	25.726	280	288.2690	Cyanidin	C_15_H_11_O_6_
4	32.789	280, 240	301.1448	Chrysoeriol	C_16_H_12_O_6_
5	34.169	280, 230, 260	303.2535	Quercetin	C_15_H_10_O_7_
6	34.939–35.260	280, 210	111.1173	Catechol	C_6_H_6_O_2_
			199.1697	Syringic acid	C_9_H_10_O_5_
			287.2202	Luteolin	C_15_H_10_O_6_
